# Clinical and epidemiological aspects of scorpionism in the interior of the state of Bahia, Brazil: retrospective epidemiological study

**DOI:** 10.1590/1516-3180.2018.0388070219

**Published:** 2019-07-15

**Authors:** Érica Assunção Carmo, Adriana Alves Nery, Carlito Lopes Nascimento, Cezar Augusto Casotti

**Affiliations:** I MSc. Nurse and Doctoral Student, Postgraduate Program on Nursing and Health, Universidade Estadual do Sudoeste da Bahia (UESB), Jequié (BA), Brazil.; II PhD. Nurse and Professor, Department of Health II and Postgraduate Program on Nursing and Health, Universidade Estadual do Sudoeste da Bahia (UESB), Jequié (BA), Brazil.; III PhD. Doctor and Professor, Department of Health and Postgraduate Program on Public Health, Universidade Estadual de Feira de Santana (UEFS), Feira de Santana (BA), Brazil.; IV PhD. Dentist and Professor, Department of Health I and Postgraduate Program on Nursing and Health, Universidade Estadual do Sudoeste da Bahia (UESB), Jequié (BA), Brazil.

**Keywords:** Accidents, Scorpions, Epidemiology, Morbidity

## Abstract

**BACKGROUND:**

Scorpion accidents have gained great visibility around the world because of the high frequency and severity with which they occur, and have become a global medical-sanitary problem.

**OBJECTIVE:**

The aim of this study was to describe the sociodemographic, clinical and epidemiological profile of scorpionism in the municipality of Jequié, Bahia, Brazil, from 2007 to 2015.

**DESIGN AND SETTING:**

Retrospective epidemiological study in the municipality of Jequié, Bahia, Brazil.

**METHODS:**

This study was based on data collected from the epidemiological investigation notification forms of the injury information system.

**RESULTS:**

There was an increase in the coefficient of incidence of scorpion accidents in Jequié from 23.4/100,000 in 2007 to 413.6/100,000 in 2015. There were 3565 cases: 54.9% were female, 58.8% were aged 20-59 years, 63.5% had brown skin color and 48.6% had incomplete primary education. Most accidents occurred in urban areas (93.1%). Homes were the main place of occurrence (84.5%) and upper limbs were the commonest sting sites (53.0%). Regarding clinical aspects, 66.4% of the cases received hospital assistance within one hour after the bite, 84.1% presented mild severity, 97.1% had local manifestations and 10.2% had systemic symptoms. Serum therapy was administered in 17.3% of the cases, and 99.9% evolved to cure.

**CONCLUSION:**

There was an increase in the incidence of scorpion accidents in the municipality, which demonstrates the need for investment in actions that reduce the morbidity and mortality caused by these accidents, such as educational campaigns and improvements in socioeconomic and health conditions.

## INTRODUCTION

Accidents caused by venomous animals have undeniable importance within public health. Among these accidents, those caused by scorpions are gaining great visibility around the world, due to the high frequency and severity with which they occur, and have become a global medical-sanitary problem.^1^

Every year, an estimated 1.5million cases and an estimated 2,600 deaths due to scorpionism occur worldwide.^2^ In Brazil, between 2000 and 2012, there was an increase of 323% in the incidence rate and 475% in mortality due to scorpion accidents, with an average of 19.6 accidents and 0.030 deaths per 100,000 inhabitants.^3^

In Brazil, wide territorial distribution of occurrences has been observed, with emphasis on the northeastern region, which has the highest rates of incidence and mortality. The state of Bahia accounts for more than 30% of the notifications in this region and has the highest average annual mortality rates in this country.^4^^,^^5^

Most scorpion species have specific habitat and microhabitat requirements, along with predictable ecological and biogeographic patterns. However, some species have high ecological plasticity and irregular distribution, which favors their occupation of environments that have been disturbed or modified by man. These species find shelter and food near and/or inside human dwellings.^6^

In view of this, and since scorpionism is also a social problem, investigation of this phenomenon at the microregional and local levels becomes important, especially through the use of techniques that can identify areas and social groups of greater risk. A further justification for conducting such investigations is the current lack of studies showing the aspects of scorpion accidents in small urban centers, where these events are quite frequent and access to health services is limited.

## OBJECTIVE

In the light of the present situation, the aim of this study was to describe the sociodemographic, clinical and epidemiological profile of scorpionism in the municipality of Jequié, Bahia, Brazil, from 2007 to 2015.

## METHODS

This was a retrospective epidemiological study on scorpion accidents reported in the municipality of Jequié, Bahia, Brazil.

The study population was composed of all of the cases of accidents involving scorpion stings that were reported at the Prado Valadares General Hospital (PVGH) between 2007 and 2015. Thishospital was chosen as the data-gathering site because it is the only serum dispensing unit in Jequié and is therefore the reference point for hospital care in cases of scorpionism in this municipality.

The data were collected directly from the epidemiological investigation sheets of the notifiable health hazard information system (SINAN, Sistema de Informação de Agravos de Notificação) of the Ministry of Health, which was made available by the Hospital Epidemiology Center of the PVGH. These records relate to investigations on all accidents involving venomous animals. For the present study, only those relating to accidents involving scorpion stings among people living in the municipality of Jequié, Bahia, were selected.

The information in the records formed three blocks of variables that were analyzed: sociodemographic characteristics (sex, age, color/race, schooling, occupation and area of residence); accident characteristics (place of occurrence, area of occurrence, month of occurrence, location of sting and time that elapsed between being stung and receiving hospital care); and clinical factors (local manifestations, systemic manifestations, classification of severity, serum therapy and evolution).

The analysis on the temporal evolution of the notifications was based on coefficients of annual incidence. These were obtained by dividing the absolute number of scorpion accidents reported by the size of the population at risk for each year of the study. The coefficients were expressed as the number per group of 100,000 inhabitants.

The population data that were used to calculate the coefficients were derived from the 2010 demographic census and from inter-census projections (2007 to 2009, 2011 and 2012) and population estimates (2013 to 2015) produced by the Brazilian Institute for Geography and Statistics (IBGE). The website of the Department of Informatics of the National Health System (DATASUS) of the Ministry of Health was also a source of data.

In calculating the coefficients, spreadsheets within Microsoft Excel 2010 were used for data tabulation and the Statistical Package for the Social Sciences (SPSS) software, version 21.0, was used for data analysis.

This study was submitted for assessment to the research ethics committee of the State University of the Southwest of Bahia, Jequié Campus, and was approved under report no. 1,376,751, on December 18, 2015. Because these data were secondary in nature, an exemption from the need to use a free and informed consent form was requested and approved.

## RESULTS

**Figure 1.**
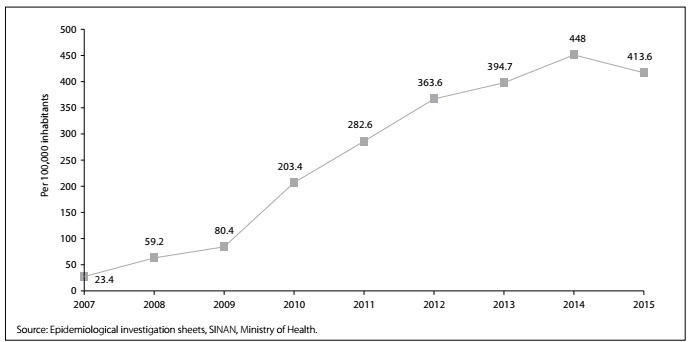
Evolution of the coefficients of incidence of scorpion accidents in the municipality of Jequié, Bahia, Brazil, 2007 to 2015.

**Figure 2.**
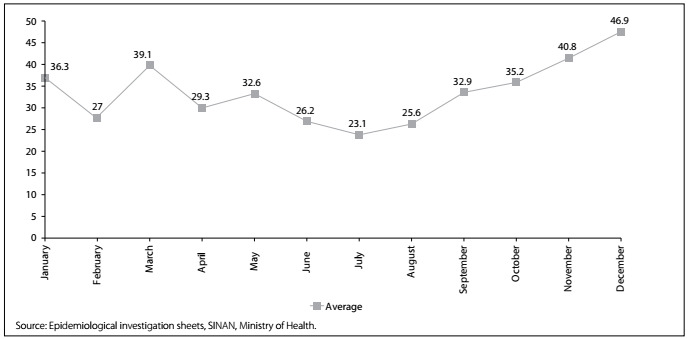
Average monthly number of scorpion accidents reported in the municipality of Jequié, Bahia, Brazil, 2007 to 2015.

Over the period from 2007 to 2015, 3565 cases of scorpionism were reported in the municipality of Jequié (state of Bahia, BA), with the highest number of occurrences in 2014 (n = 722). Therewas an increase in the incidence of notifications of scorpionism in the municipality (Figure 1), from 23.4/100,000inhabitants in 2007 to 413.6/100,000 inhabitants in 2015, with a peak in 2014 (448.0/100,000 inhabitants). Analysis on the monthly distribution showed that there was no pattern of concentration of accidents between the months. Themonthly average number of accidents ranged from 23.1cases in July to 46.9 in December (Figure2).

The victims (Table 1) were predominantly female (54.9%), aged between 20 and 59 years (58.8%), of brown skin color (63.5%), with incomplete primary education (48.6%) and living in the urban area (94.8%). In relation to occupation, 29.0% were minors or only students.

**Table 1.** Characterization of the cases of scorpionism according to sociodemographic variables. Jequié, Bahia, Brazil, 2007 to 2015

Variables	n	%
Sex (n = 3,563)		
Male	1,608	45.1
Female	1,955	54.9
Age range (years) (n = 3,556)		
0 to 9	365	10.3
10 to 19	616	17.3
20 to 59	2,092	58.8
60 or over	483	13.6
Color/race (n = 2,955)		
White	480	16.2
Black	581	19.7
Brown	1,876	63.5
Asian	14	0.5
Indigenous	4	0.1
Schooling (n = 2,318)		
Completed higher education	52	2.2
Incomplete higher education	55	2.4
Full high school	435	18.8
Incomplete high school	237	10.2
Completed elementary education	73	3.2
Incomplete elementary school	1,127	48.6
Illiterate	339	14.6
Occupation (n = 2,572)		
Minor/student	746	29.0
Domestic	555	21.6
Working in commerce	249	9.7
Rural worker	103	4.0
Mason	140	5.4
Retired	211	8.2
Others	568	22.1
Area of residence (n = 3,555)		
Urban	3,371	94.8
Rural	184	5.2

Source: Epidemiological investigation sheets, SINAN, Ministry of Health.

Accidents occurred more frequently in the urban area (93.1%), and the victim’s home was the main place of occurrence (84.5%). Regarding the location of the bite, the upper limbs were the body segments most affected, accounting for 53.0% (Table 2).

Regarding clinical factors, as described in Tables 2 and 3, local manifestations were shown in 97.1% of the injured individuals, with emphasis on pain, paresthesia and edema, respectively, in 92.4%, 20.3% and 18.6% of the cases. Systemic manifestations were observed in 10.2% of the victims, and the most frequent of these were hypertension (4.7%), nausea/vomiting (1.3%) and headache (1.1%).

**Table 2.** Description of the cases of scorpionism, according to the characteristics of the accident and the clinical characteristics of the victims. Jequié, Bahia, Brazil, 2007 to 2015

Variables	n	%
Occurrence zone (n = 3,530)		
Urban	3,286	93.1
Rural	244	6.9
Place of occurrence (n = 2,826)		
Residence	2,387	84.5
Third-party house	28	1.0
Public highway	68	2.4
Farm	86	3.0
In the workplace	205	7.3
Others	52	1.8
Location of the bite (n = 3,423)		
Upper limbs	1,815	53.0
Lower limbs	1,296	37.9
Others	312	9.1
Local manifestations (n = 3,528)		
No	103	2.9
Yes	3,425	97.1
Systemic manifestations (n = 3,226)		
No	2,897	89.8
Yes	329	10.2
Time elapsed until hospital care (3,243)		
< 1 hour	2,154	66.4
1 to 3 hours	677	20.9
> 3 hours	412	12.7
Severity classification (n = 3,465)		
Mild	2,913	84.1
Moderate	481	13.9
Severe	69	2.0
Serum therapy (n = 3,430)		
No	2,836	82.7
Yes	594	17.3
Evolution (n = 3,214)		
Cure	3,211	99.9
Death due to scorpionism	3	0.1

Source: Epidemiological investigation sheets, SINAN (Sistema de Informação de Agravos de Notificação), Ministry of Health.

**Table 3.** Clinical manifestations observed among victims of scorpionism. Jequié, Bahia, Brazil, 2007 to 2015

Manifestations	n	%
Local manifestations		
Pain	3,293	92.4
Paresthesia	723	20.3
Edema	665	18.6
Bruise	24	0.7
Others	223	6.2
Systemic manifestations		
Vomiting/nausea	48	1.3
Headache	39	1.1
Dizziness	27	0.7
Sweating	9	0.3
Arterial hypertension	167	4.7
Arterial hypotension	8	0.2
Tachycardia	5	0.1
Dyspnea	7	0.2
Others	32	0.9

Source: Epidemiological investigation sheets, SINAN (Sistema de Informação de Agravos de Notificação), Ministry of Health.

The time that elapsed between being bitten and receiving hospital care was mostly less than one hour (66.4%). Most of the cases (84.1%) were classified as mild, while 2.0% presented a severe clinical picture. Serum therapy was administered in 17.3% of the cases, and 99.9% evolved to cure.

## DISCUSSION

Over the period analyzed, there was an alarming increase in the coefficient of incidence of scorpion accidents in the municipality of Jequié (23.4/100.000 to 413.6/100.000). This was higher than the increase that has been estimated for all of Brazil: between 2001 and 2012, the accident rate went from 10.5/100,000 to 32.3/100,000.^4^ The coefficient for Jequié also differs from what has been found in other municipalities in this country, such as in Campina Grande, state of Paraíba, from 2007 to 2012^7^ and in Belo Horizonte, state of Minas Gerais, from 2005 to 2009,^8^ where there were declines in the coefficient, from 132.0/100,000 to 108.0/100,000 and from 28.6/100,000 to 24.3/100,000, respectively.

It should be noted that in the present study, the growth shown may reflect not only the increase in the number of cases, but also improvements in the notification process. The coefficient increased in 2009, when the Hospital Epidemiology Center of the PVGH was implemented. Through this, compulsory notifications of diseases and health hazards began to be carried out in a judicious manner. In addition, increased awareness within the population regarding the emergency nature of these accidents may have given rise to greater demand for healthcare services, and thus may have increased the numbers of notifications.

Regarding the monthly distribution, it was observed that the accidents did not present seasonal behavior, and a certain degree of uniformity of occurrence of this health hazard over the months of the year was seen. This finding can be explained by the fact that Jequié presents environmental conditions that are favorable for survival and proliferation of scorpions throughout the year, such as ideal temperatures, humidity and abundant food. This differs from the situations found in the state of Pernambuco^9^ and in the city of Belo Horizonte,^8^ and in countries such as Iran^10^ and Tunisia,^11^ where scorpion accidents have been reported to be more frequent in the hotter and rainier periods of the year.

The process of urbanization has been reported to be a factor contributing towards scorpionism. This was also observed in the present study, since 94.8% of the accidents occurred in urban zones, as seen in other studies conducted in different regions of Brazil ^7^^,^^12^ and in Sudan.^13^ The urbanization of scorpionism has been explained as being due to disordered urban growth, the inadequacies of infrastructure and the environmental imbalance.^14^ Uncontrolledincreases in the urban population lead to occupation of irregular areas, with severe infrastructure problems such as lack of basic sanitation and poor housing conditions. Theseare factors that favor availability of shelter and proliferation of these animals.^15^ In addition, the ease of adaptation of scorpions to changes in the environment, in combination with difficulties in implementing preventive programs within the population, boost the risks of occurrence of such accidents.^16^

The higher frequency of accidents suffered by females corroborates other surveys conducted in Brazil ^l7^^,^^12^^,^^17^ and in other countries.^10^^,^^18^ The fact that the highest frequency of accidents was at home (85.5%), together with the high proportion of cases in which the victim’s occupation related to domestic activities, provides emphasis in explaining why the proportion of women affected was greater. Females in this setting might have greater exposure related to domestic activities, such as cleaning of places that often serve as shelter for scorpions, such as sinks, bathroom drains, clothing and shoes.^7^

Nevertheless, other studies have pointed out higher occurrence of male scorpionism.^9^ Activities performed outside the home, especially those relating to construction and agriculture, have been reported to be related to scorpion accidents.^16^

The high proportion of the scorpion bite victims of the present study that were classified as having the sociodemographic characteristic of low schooling corroborates the findings of a previous study on venomous animal poisoning in Brazil.^4^ That study showed that there were negative correlations between scorpionism and literacy and the Human Development Index (HDI). These findings contribute towards the hypothesis that socially and economically disadvantaged populations present greater vulnerability to scorpion accidents,^3^ considering that schooling contributes towards better socioeconomic conditions.

The location of the bite has been reported to be one of the factors that influence the severity of cases, such that the closer it is to vital organs, the greater the complications and possibilities of side effects will be.^19^ In the present study, it was found that the upper limbs were the body segments most affected, similar to what was found in other studies.^9^^,^^12^^,^^18^

This result shows that scorpion bites usually occur while household chores are being done,^8^^,^^12^ or when the victims are putting on their clothes or shoes,^12^ or while working in environments that are considered to provide suitable shelters for these animals, without using personal protective equipment (PPE) such as gloves and boots.

Another matter that deserves attention is early treatment for injured people. If it is necessary to apply therapeutic serum, this should be administered as soon as possible, so that the venom is immediately neutralized. In the present study, the largest proportion of the victims were hospitalized less than one hour after the sting, similar to what was observed in studies conducted in northeastern Brazil,^7^ the state of Ceará^12^ and the city of Belo Horizonte.^8^ From the point of view of epidemiological surveillance, this finding may mean that there were improvements to access to information concerning the need for urgent medical services in cases of scorpion stings.^8^ The findings from the present study indicate that the healthcare service in this region is providing relatively good medical care for the victims of scorpionism.

Furthermore, the fact that attendance was provided quickly may explain why a greater proportion of the cases were classified as mild. This may also explain why most of the accidents did not present systemic manifestations, i.e. they did not present signs and symptoms that would be indicative of greater severity. On the other hand, 97.1% of the cases presented local manifestations, with emphasis on pain, paresthesia and edema. Theseclinical characteristics are similar to those found in studies conducted in different Brazilian states ^12^^,^^20^ and in regions of Iran^21^ and Saudi Arabia.^22^

In general, cases with mild symptoms require simple therapeutic measures such as administration of analgesics and antihistamines. However, in cases with systemic symptoms, use of antivenom and other measures against anaphylactic reactions is recommended.^18^ In the present study, the number of cases that received serum therapy did not match the proportion of systemic manifestations. This finding may have been due to other criteria considered in determining that this treatment should be used, or due to possibly unnecessary use of serum.

Regarding case evolution, the clinical profile found may explain the high proportion of cases that were cured, along with the relatively low number of deaths. The description of the deaths corroborates what has been pointed out in the literature. Thus, children have been shown to present greater susceptibility to scorpion toxin, especially those under nine years of age.^11^^,^^23^ Moreover, a relationship between the time that elapses from receiving the bite to receiving hospital care and the prognosis for the case has been demonstrated.^9^

The use of secondary data can be highlighted as a limitation of this study. The main disadvantages of using secondary data are that variables may be under-registered and cases may be underreported. These situations can be generated both through failure to complete the notification forms and through occurrences of cases with mild symptoms for which healthcare services are not sought. Hence, such situations may lead to underestimation of the numbers of accidents and make it difficult to characterize the cases.

However, despite such limitations, the SINAN system of the Brazilian Ministry of Health was seen to be an important tool for conducting epidemiological studies, given that it is the official database for registering diseases and health hazards in Brazil.

## CONCLUSION

This study showed that the coefficient of incidence of scorpion accidents in the municipality of Jequié has increased. The clinical and epidemiological profile of the scorpionism cases in this study corroborates what has been found in other surveys in Brazil and in other countries, i.e. predominance of scorpionism in urban areas, among the economically active age group with low schooling levels. The importance of early hospital care for better evolution of cases was also observed.

These findings indicate that multidisciplinary actions involving different healthcare sectors, environmental management and the population itself are essential for reducing morbidity and mortality due to scorpionism. Among such actions, the most important of these are investment in educational campaigns, improvements in socioeconomic and health conditions, and improvements in the hospital care provided for bite victims, so as to ensure immediate care.
